# Enhancement of the Electric-Force Response of Carbon Black/Silicone Rubber Composites by Silane Coupling Agents

**DOI:** 10.3390/molecules29122740

**Published:** 2024-06-08

**Authors:** Yanfang Zhao, Yang Yang, Bangwei Wan, Tianyu Ding, Xun Sha

**Affiliations:** 1 Faculty of Civil Engineering and Mechanics, Kunming University of Science and Technology, Kunming 650500, China; z18587206717@163.com (Y.Z.); 20212210041@stu.kust.edu.cn (B.W.); 15145498538@163.com (T.D.); shaxun@stu.kust.edu.cn (X.S.); 2 Yunnan Key Laboratory of Disaster Reduction in Civil Engineering, Kunming 650500, China; 3 International Joint Laboratory for Green Construction and Intelligent Maintenance of Yunnan Province, Kunming 650500, China

**Keywords:** carbon black, silane coupling agents, silicone rubber, structural health monitoring, shoulder peak

## Abstract

Flexible strain sensors have a wide range of applications in the field of health monitoring of seismic isolation bearings. However, the nonmonotonic response with shoulder peaks limits their application in practical engineering. Here we eliminate the shoulder peak phenomenon during the resistive-strain response by adjusting the dispersion of conductive nanofillers. In this paper, carbon black (CB)/methyl vinyl silicone rubber (VMQ) composites were modified by adding a silane coupling agent (KH550). The results show that the addition of KH550 eliminates the shoulder peak phenomenon in the resistive response signal of the composites. The reason for the disappearance of the shoulder peak phenomenon was explained, and at the same time, the mechanical properties of the composites were enhanced, the percolation threshold was reduced, and they had excellent strain-sensing properties. It also exhibited excellent stability and repeatability during 18,000 cycles of loading–unloading. The resistance-strain response mechanism was explained by the tunneling effect theoretical model analysis. It was shown that the sensor has a promising application in the health monitoring of seismic isolation bearings.

## 1. Introduction

Structural health monitoring (SHM) of seismic isolation bearings plays a vital role in ensuring the health and safety of bridges [1]. Evaluating the health of bridge structures is necessary to reduce repair costs, maintenance costs, and to demonstrate structural safety [2,3]. The sensing system is the key to realizing the health monitoring of bridge structure, and the traditional metal material strain sensors have the disadvantages of poor tensile ability, limited application range, and difficulties in continuous monitoring [4]. Therefore, the preparation of a flexible strain transducer with good stability and a monotonic resistance response signal is crucial for the health monitoring of seismic isolation bearings.

Conductive polymer composite (CPC) flexible strain sensors have received much attention in recent years. CPC strain sensors consisting of a conductive filler and a polymer matrix have been widely used for human motion monitoring [5,6,7], structural health monitoring [8,9], and health care [10,11]. Conductive filler is an important component in the preparation of flexible strain sensors. The use of metal nanowires [12] and nanoparticles [13] as conductive fillers can significantly improve the electrical conductivity of composites, but the interaction between the rubber matrix and these fillers is weak, resulting in poor mechanical properties. In addition, these fillers are expensive, increasing the cost of the material. Conductive carbon materials (carbon nanotubes (CNTs) [14], graphene (GR) [15], carbon black (CB) [16], etc.) have the advantages of light weight, flexibility and high conductivity and are ideal candidates for the preparation of conductive polymer composites with excellent overall performance. Flexible polymers with high elongation at the break, such as polydimethylsiloxane (PDMS) [17,18], thermoplastic polyurethane (TPU) [19,20], natural rubber (NR) [21], and methyl vinyl silicone rubber (VMQ) [7,22], are usually chosen as the matrix materials. Among them, methyl vinyl silicone rubber (VMQ) has excellent resilience and very good aging resistance and is often used as a substrate for flexible strain sensors.

Compared with other conductive fillers, CB has the advantages of a large, specific surface area, low cost, and good reinforcing properties, and it is widely used in the preparation of flexible conductive composites. For example, Huang et al. [23] used ultrasound-assisted dip coating to prepare three-dimensional, lightweight piezoresistive-sensing materials by coating PUs with a mixture of carbon nanotube CNTs and CB. The sensor exhibits fast response (65 ms), instant recovery, and excellent stability and repeatability. Zheng et al. [24] prepared CNT-CB/PDMS flexible strain sensors using solution mixing and casting molding. The results show that the sensor exhibits high stretchability and a wide-strain sensing range (~300% strain). It exhibits excellent repeatability, good stability, and excellent durability (2500 cycles at 200% strain) in stretch–release cycles. However, the phenomenon of shoulder peak was observed in the resistance response signals of the above studies. The shoulder peak phenomenon is the nonmonotonic resistance change signal of the sensor during cyclic loading and unloading. The peak that occurs when the strain reaches its maximum is the main peak, and the peak that occurs during the unloading phase is the shoulder peak. This nonmonotonic resistive response signal affects the reproducibility and durability of the resistive response signal, which brings difficulties in signal processing and real-time monitoring and severely limits the practical application of strain sensors in structural health monitoring. Therefore, it is important to eliminate the shoulder peak phenomenon and prepare highly stable sensors for the health monitoring of seismic isolation bearings. 

Conductive fillers tend to aggregate due to their own van der Waals forces, further exacerbating the difficulty of uniformly dispersing conductive materials in an elastic matrix. Silane coupling agents have low surface energy and can be evenly distributed on the treated surface, thus improving compatibility and dispersion between dissimilar materials. Han et al. [25] used a silane coupling agent (KH570) to modify CNT to fabricate CNT/PDMS composites via a continuous thermal spinning strategy. Through the KH570 modification of CNT modification, the aggregation between CNTs was effectively reduced so that the CNTs could be uniformly dispersed in the PDMS matrix. The composites have the advantages of a wide sensing range (0–100%), excellent fatigue resistance, high linearity and good mechanical properties, and good tensile repeatability (50% strain for 1000 cycles). Chen et al. [26] prepared CNTs-KH550/PDMS composites with dual-sensing layers using a simple and scalable coating method, one sensing layer being a CNT-PDMS conductive layer and the other sensing layer being an ultrasensitive conductive layer with a multicracked structure composed of CNTs and a silane coupling agent (KH550). Since the crack-expanded sensing mechanism of this sensor dominates the sensing behavior of the CNT-KH550 layer, it thus exhibits the advantages of an ultra-low sensing range (0.01%), a fast response time (68 ms), and a good reproducibility of 10,000 stretch–release cycles. In summary, the silane coupling agent helps the conductive filler to be uniformly dispersed in the matrix and construct a more organized conductive network. Therefore, KH550 is used to modify CB, and the modified CB contains a large number of alkoxyl groups, which helps CB to be uniformly dispersed in VMQ through hydrogen bonding, improving the compatibility between CB and VMQ, and forming a more ordered and tightly packed conductive network, which improves the stability and durability of the strain sensor.

In this study, CB/VMQ and CB-KH550/VMQ conductive composites were prepared with a solution method using silane coupling agent (KH550)-modified carbon black (CB) as conductive filler and silicone rubber (VMQ) as a matrix. The dispersion of the conductive fillers in the VMQ matrix was analyzed using SEM images and EDS energy spectra. FTIR and Raman were utilized to explore the interfacial interaction between the filler and matrix. In addition, the effect of the synergistic effect of CB and KH550 on the resistance-strain response properties of the composites under cyclic strain was also investigated. The reason for eliminating the shoulder peak phenomenon is explained, and a theoretical model for quantitatively analyzing the corresponding properties of the force electrical force is established, which is helpful for conductive composites for health monitoring of seismic isolation bearings.

## 2. Results and Discussion

### 2.1. Mechanical Properties of Two Composites

**Figure 1:**
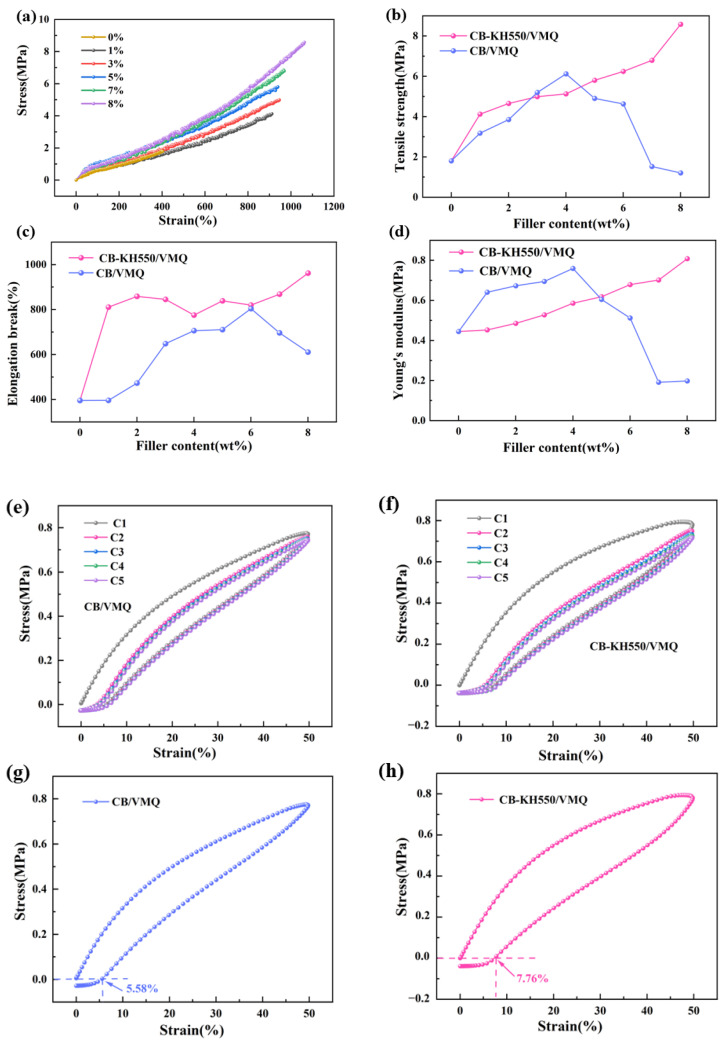
(**a**) Stress–strain curves of CB-KH550/VMQ composites; (**b**–**d**) tensile strength, elongation at break and Young’s modulus of CB/VMQ and CB-KH550/VMQ composites; (**e**,**f**) cyclic stress–strain curves of CB/VMQ and CB-KH550/VMQ composites at 50% strain; (**g**,**h**) first cycle stress–strain curves of CB/VMQ and CB-KH550/VMQ composites first cyclic stress–strain curves.

Figure 1a illustrates the stress–strain curve of the CB-KH550/VMQ composite. It is clearly seen that a good linear relationship between stress–strain is presented regardless of the CB content. As the CB content increases, the slope of the curve increases, proving that the stiffness of the composite increases. Figure 1b demonstrates the tensile strength of the two composites, and the results show that the tensile strength of CB-KH550/VMQ composite increases with the increase in filler, and the maximum value is 8.58 MPa, which is 1.4 times more than that of CB/VMQ composite. In CB/VMQ composites, when the CB content reaches 4%, the tensile strength decreases gradually with the increase in CB content, which is due to the agglomeration of CB particles that impedes the continuity of CB in the VMQ matrix. In CB-KH550/VMQ composites, on the other hand, the surface modification of KH550 enhances the compatibility and dispersion of CB with the VMQ matrix, the CB content increases, and the CB can be uniformly dispersed in the matrix, so the tensile strength shows a monotonically increasing trend. From Figure 1c, it can be seen that the elongation at the break of both CB-KH550/VMQ composites is higher than that of CB/VMQ composites. The elongation at the break of CB-KH550/VMQ composites is higher than that of CB/VMQ composites, as seen in Figure 1c. Figure 1d shows Young’s modulus of the two composites, and it is clearly seen that, with the increase in the conductive filler content, Young’s modulus of the CB-KH550/VMQ composite rises to 0.81 MPa, which is an increase of 6.4% compared with that of the CB/VMQ composite, indicating that the resistance to deformation of the CB-KH550/VMQ composite is improved. The KH550-modified CB contains a large number of alkoxyl groups, which helps the CB to be uniformly dispersed in the VMQ through hydrogen bonding. Figure 1e,f, demonstrate the cyclic stress–strain curves for both composites at 50% strain. A clear hysteresis loop is observed in the first cycle (C1) curve, and a large amount of residual strain is present. From the first cycle (C1) to the second cycle (C2), the stress and hysteresis return line area decreases. Figure 1g,h, show the residual strain of the composite after the first loading–unloading. The resilience of the composite is evaluated by $\mathrm{ER}=\frac{\varepsilon -\varepsilon_{R}}{\varepsilon }\times 100\%$, where ε is the strain, and ε_R_ is the residual strain after the first cycle of unloading. The results show that the residual strain of the composites modified by KH550 increases, and the ER of the composites decreases from 94.42% to 92.24%, which is due to the increase in the stiffness of the composites modified by KH550, so the ER decreases.

### 2.2. Strain Sensing Properties of Composite Materials

#### 2.2.1. Resistance-Strain Response under Static Loading

**Figure 2:**
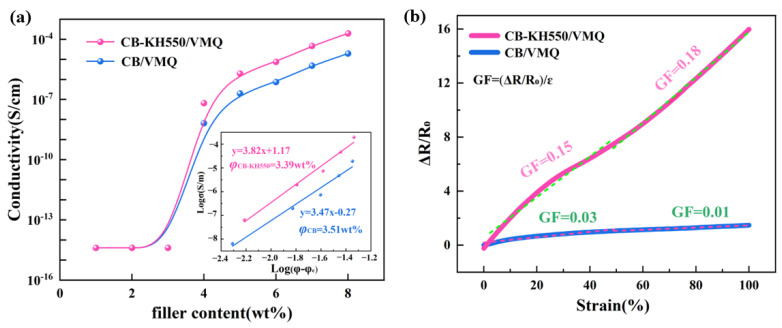
(**a**) Electrical conductivity of the two composites versus nanofiller content; (**b**) resistance-strain response curves and GF values of the two composites at a strain of 100%.

Figure 2a demonstrates the typical percolation behavior of the two composites as the bulk conductivity increases with the increase in conductive fillers [27]. According to the statistical percolation theory [28], the relationship between the electrical conductivity of the composite and the conductive filler is shown in the following equation:

(1) $$\sigma ={\;\sigma }_{0}{(\varphi \;-{\;\varphi }_{c})}^{t},$$

In Equation (1), σ is the volumetric conductivity of the composite at a certain nanofiller content, σ_0_ is the scale factor, φ is the mass fraction of nanofiller in the conductive composite, φ_c_ is the percolation threshold of the conductive composite, and t is the dimensionality of the conductive network, which is 1.3 and 2.0 for the two- and three-dimensional conductive networks, respectively. The φ_c_ of CB/VMQ and CB-KH550/VMQ composites are 3.51 wt% and 3.39 wt%, respectively. It is clearly seen that the use of KH550-modified CB filler reduces the percolation threshold of the composites by 3.4% as compared to CB/VMQ composites. Based on the linear relationship of the fitted data of CB/VMQ and CB-KH550/VMQ, the composites in Figure 2a yield t 3.48 and 3.82, respectively, which proves that both composites form a three-dimensional tunneling conductive network [29].

To avoid large agglomeration of fillers and to ensure good sensing performance of the sensors, two composites with a CB content of 6% were selected to analyze the resistance–strain response curves (∆R/R_0_, where R_0_ is the initial resistance, R is the test resistance, and ∆R = R − R_0_) under static loading as well as the two phases of the linear fitting (0–50%, 50–100%), as shown in Figure 2b. The sensitivity of the composites was evaluated using GF = (ΔR/R_0)_/ε, with ε being the strain. It is clearly seen that the resistance of the two composites increases linearly with the increase in strain; in addition, the GF values of the two composites are lower, which is because the two composites undergo localized deformation and destruction of the conductive network only in the linear response region, which hinders the formation of effective conductive channels and leads to their lower sensitivity.

#### 2.2.2. Resistance-Strain Response under Dynamic Loading

**Figure 3:**
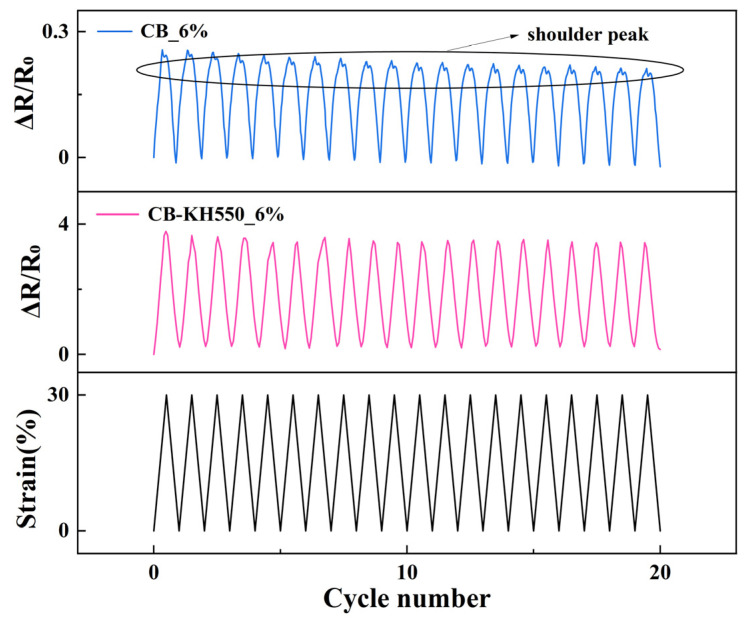
Resistance-strain response of composites at 30% strain.

The dynamic resistance-strain response properties of the two composites are demonstrated in Figure 3, respectively, and it is clearly seen that there is an obvious shoulder phenomenon in CB/VMQ composites, and the shoulder phenomenon is prevalent in conductive polymer composites [7,21,30,31]. The shoulder effect was significantly eliminated by KH550-modified composites.

**Figure 4:**
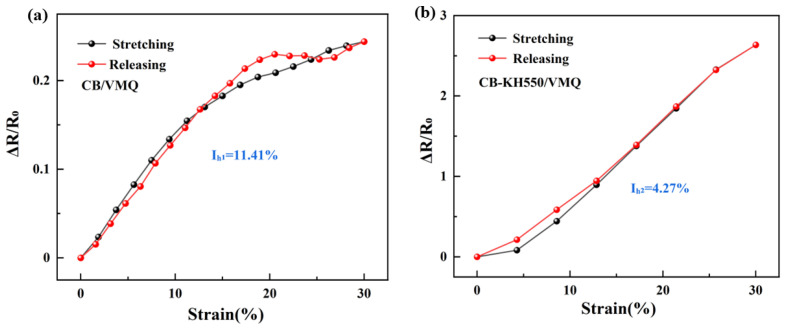
(**a**,**b**) Resistance-strain response of CB/VMQ, CB/VMQ composites at strain of 30% for the 8th cycle of loading–unloading.

To explain the shoulder peak phenomenon, the resistance–strain curves in the 8th cyclic loading of two composites were selected from Figure 3. In Figure 4a, it is seen that the resistance of CB/VMQ composites shows a clear upward trend with increasing strain. This is because as the strain increases, the CB packing spacing that constitutes the conductive network increases, leading to the continuous destruction of the conductive network, and the resistance increases and strain unloading, the conductive network reconstruction, and the resistance decreases. At the same time, due to the viscoelasticity of the matrix [22], the conductive network continues to be disrupted, and the phenomenon of shoulder peaks occurs. Therefore, the reason for the shoulder peak phenomenon is considered to be the competition between the destruction and reconstruction of the conducting network during cyclic loading–unloading and the viscoelastic nature of the matrix [30]. However, as shown in Figure 4b, the CB-KH550/VMQ composite maintains a linear increase during loading, and a monotonic decrease during unloading without shoulder peaks. To further explain the shoulder peak phenomenon, the area of the region of the hysteresis effect of the composite was evaluated by using $I_{H}=\frac{\left|A_{S}-A_{R}\right|}{A_{S}}$, where A_S_ and A_R_ are the areas of tension and unloading in the resistance–strain curves, respectively. I_H1_ and I_H2_ are the areas of the region of hysteresis effect during the stretch-unloading process of CB/VMQ, and CB-KH550/VMQ composites, respectively. The results show that the I_H2_ of CB-KH550/VMQ composites is lower than the I_H1_ of CB/VMQ composites, and the larger the value of I_H_, the more obvious the hysteresis effect is, the higher the residual resistance is, and the worse the resistive response signal recovery is. Therefore, CB modified by KH550 forms a more compact conductive network, which helps to reduce the hysteresis effect, thus eliminating the shoulder peak phenomenon.

**Figure 5:**
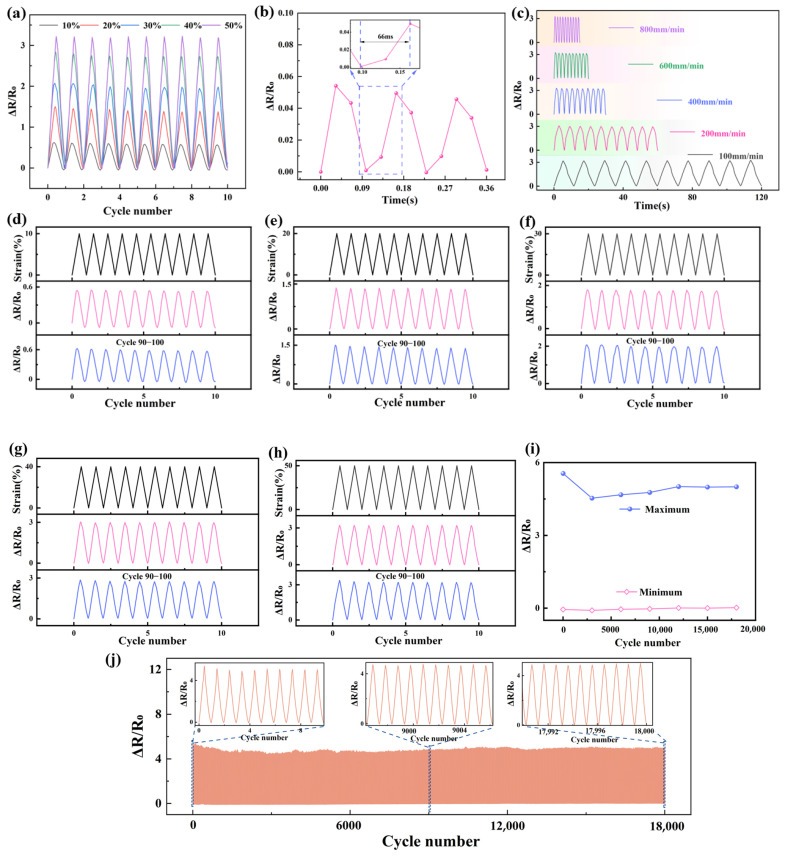
(**a**) Resistive response of CB-KH550/VMQ composites at different strains; (**b**) response time of CB-KH550/VMQ composites at a strain of 1% and a rate of 200 mm/min^−1^; (**c**) resistive response of CB-KH550/VMQ composites at different rates; (**d**–**h**) resistive response of CB-KH550/VMQ composites for 100 cycles at strains of 10%, 20%, 30%, 40%, and 50%; (**i**) statistics of the maximum and minimum resistive values of CB-KH550/VMQ composites for 18,000 tensile–release cycles at a strain of 50%; and (**j**) resistive response of CB-KH550/VMQ composites for 18,000 cycles at a strain of 50%.

Figure 5a demonstrates the resistance–strain response curve of the CB-KH550/VMQ composite, which exhibits excellent repeatability and stability with increasing strain. As shown in Figure 5b, the CB-KH550/VMQ composite obtains a response time of 66 ms at a small strain of 1% and a tensile rate of 200 mm/min, which is faster compared to other composites [25,32,33,34,35], and the shorter response time means that the composites can respond to the external stimuli quickly. Meanwhile, the stabilized resistance response of strain sensors under different tensile rates is also very important in practical engineering applications. As shown in Figure 5c, the resistance-strain response of the composites at 50% tensile strain, with different rates, is investigated. It is clear that the resistance-strain response remains stable as the rate increases, confirming that the sensing behavior is not affected by the rate. Figure 5d–h demonstrates the resistance-strain response of CB-KH550/VMQ composites at 10%, 20%, 30%, 40%, and 50% strain, respectively, and it is clear that the resistance change is consistent with the strain change. The 100 cycles of loading–unloading resistance signals for different strains remain stable without significant fluctuations, demonstrating excellent stability. To further test the long-term resistance-response behavior of the CB-KH550/VMQ composite, 18,000 loading–unloading cycles at 50% strain are presented in Figure 5j. It is clearly seen that the resistance-response signal of the composite is always stable. As can be seen from the locally enlarged image, no shoulder peak phenomenon was observed in 18,000 cycles, indicating that the composite material has excellent stability and durability. The maximum and minimum values of resistance for the selected cycles (1st, 3000th, 6000th, 9000th, 12,000th, 15,000th, and 18,000th) were studied to assess the relative resistance changes, as shown in Figure 5i. The maximum resistance decreases as the number of cycles increases, which is due to the effect of hysteresis effect. After several loading–unloading cycles, the maximum and minimum resistance values change weakly and show excellent reversibility, which is because the composite is affected by pre-stretching [36].

### 2.3. Dispersion and Interface Effects of Composites

**Figure 6:**
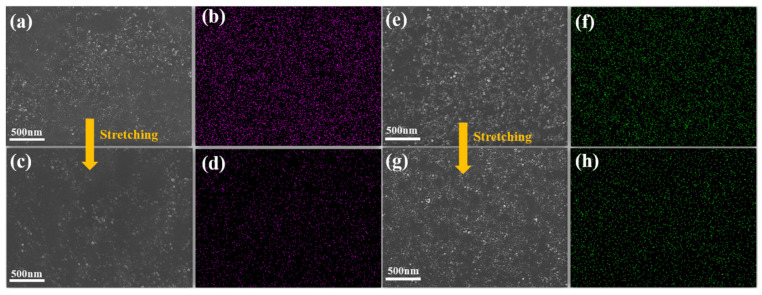
(**a**,**b**) SEM image and EDS energy spectra of CB/VMQ composites before stretching; (**c**,**d**) SEM image and EDS energy spectra of CB/VMQ composites after 50% strain stretching; (**e**,**f**) SEM image and EDS energy spectra of CB-KH550/VMQ composites before stretching; (**g**,**h**) SEM image and EDS energy spectra of CB-KH550/VMQ composites after 50% strain stretching.

Figure 6a–h show the SEM images and EDS energy spectra of CB/VMQ composites and CH-KH550/VMQ composites before and after 50% strain stretching, and the S element, which is unique to carbon black, is visible in the energy spectra. From Figure 6a–d, it can be seen that the CB in the CB/VMQ composites before stretching showed an obvious agglomeration phenomenon, and the gap between the CB increased after stretching, the conductive network was seriously damaged, and the energy spectra show that the conductive channels were obviously reduced after stretching, which is one of the important reasons for the shoulder peak phenomenon in the resistive strain-response performance. As can be seen in Figure 6e–h, the uniform distribution of S elements is clearly seen in the energy spectrograms before stretching, which confirms that the KH550-modified CB in the CB-KH550/VMQ composites is uniformly dispersed, and the conductive network is kept intact after stretching, and the energy spectrograms show that most of the conductive channels are kept intact, which is one of the important reasons for the disappearance of the shoulder peak effect.

**Figure 7:**
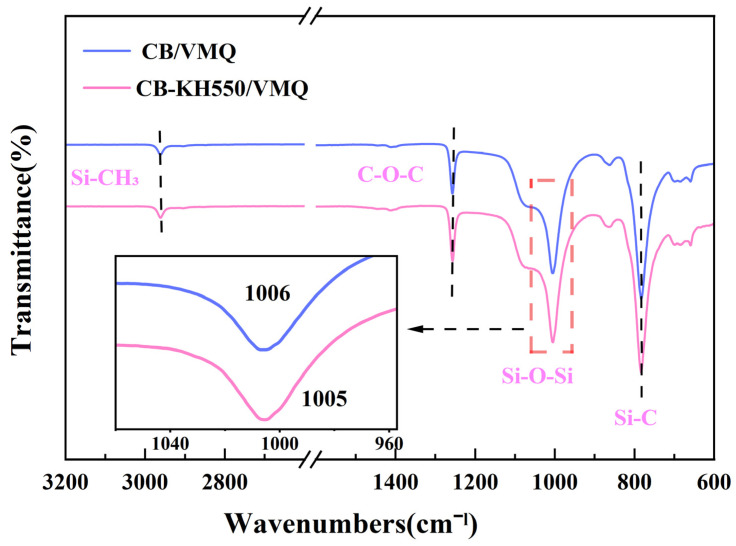
Fourier transform infrared spectroscopy (FTIR) of CB/VMQ, CB-KH550/VMQ composites.

Figure 7 shows the absorbance curves of the composites within the FTIR spectral wavelengths 600~3200 cm^−1^. The peaks at 2962 cm^−1^ and 659 cm^−1^ are attributed to the stretching vibrations of Si-CH^3^ and Si-C. The bending vibration of C-O-C produces the peak at 1257 cm^−1^. The peaks at 1006 cm^−1^ and 1005 cm^−1^ are attributed to the stretching vibration of Si-O-Si. The characteristic peak of CB-KH550/VMQ composites shifted from 1006 cm^−1^ to 1005 cm^−1^ as seen in the local zoomed-in image, which is consistent with the changes concluded in other studies [37], and the change is attributed to the formation of hydrogen bonding between the residual oxygen-containing groups of the nanofillers and the Si-O-Si groups in the silicone rubber matrix, which facilitates the compatibility. 

**Figure 8:**
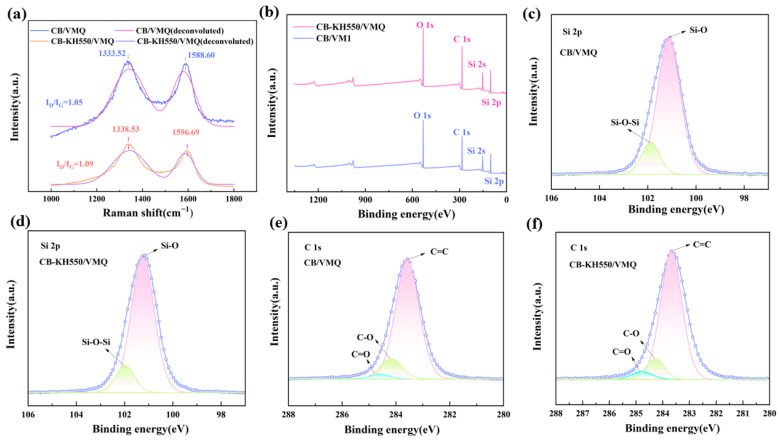
(**a**) Raman spectra of CB/VMQ, CB-KH550/VMQ composites; (**b**) XPS energy spectra of CB/VMQ, CB-KH550/VMQ composites; (**c**,**d**) Si 2p spectra of CB/VMQ, CB-KH550/VMQ composites; (**e**,**f**) C 1s spectra of CB/VMQ, CB-KH550/VMQ composites.

To further investigate the interfacial interaction between the filler and matrix, Raman spectra of CB/VMQ and CB-KH550/VMQ composites are shown in Figure 8a. The D peak is used to characterize the disordered structure and defects in carbon materials, and the G peak is attributed to the stretching vibrational mode of the bonds within the carbon atom faces, and its peak intensity is also related to defects. It can be seen that the D peak of the composite modified with KH550 is shifted from 1333.52 cm^−1^ to 1338.53 cm^−1^, and the G peak is shifted from 1588.60 cm^−1^ to 1596.69 cm^−1^, which indicates that the vibration of the D peak and the G peak of the composite is strengthened, which improves the interfacial interactions between the conductive filler and the matrix. In addition, the ratio of D peak and G peak intensities (I_D_/I_G_) can be used to evaluate the structural integrity and interfacial strength of the composites and to correctly estimate the I_D_/I_G_ ratios; the Raman spectra were deconvoluted. The maximum height values of D and G peaks were obtained with reverse convolution, and it was found that the I_D_/I_G_ values of CB/VMQ and CB-KH550/VMQ composites were 1.05 and 1.09, respectively, which were similar to those of the two composites, indicating that the KH550 did not damage the carbon structure of the composites.

The XPS spectra of CB/VMQ and CB-KH550/VMQ composites are shown in Figure 8b. The fine scans of silicon and carbon are shown in Figure 8c–f. The binding energies in the Si 2p spectra of the CB-KH550/VMQ composites changed significantly, with the Si-O bond and Si-O-Si bond shifted from 101.1 eV and 101.9 eV to 101.3 eV and 102.0 eV, respectively, indicating that the carbon black has strong interfacial interactions with the silane coupling agent. The C 1s spectrum of the unmodified composite has three peaks at 283.6 eV, 284.2 eV, and 284.7 eV, belonging to C=C, C-O, and C=O, respectively. The C=C, C-O, and C=O of the modified composite are shifted to 283.7 eV, 284.3 eV, and 284.8 eV. Similar to the results of other studies [25], the peaks of C=C, C-O, and C=O were only slightly shifted after modification with silane coupling agents, suggesting that the chemical modification of KH550 by silicon can still be detected on CB.

### 2.4. Strain Sensing Mechanism

**Figure 9:**
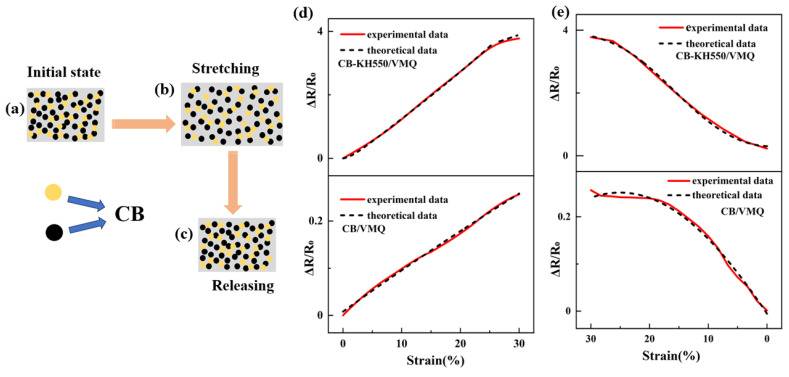
(**a**–**c**) Changes of the conductive network of the composites throughout the cycling process; (**d**) resistance of the experimental results versus the theoretical results for the tensile stage of CB-KH550/VMQ and CB/VMQ composites; (**e**) resistance of the experimental results versus the theoretical results for the unloading stage of CB-KH550/VMQ and CB/VMQ composites (black color indicates theoretical predictions, red color indicates experimental results).

In studying the resistance-strain relationship of CB-KH550/VMQ composites, we constructed a hypothetical schematic to simulate the stretch–unload process. Figure 9a demonstrates the initial state of KH550-modified CB in the VMQ matrix, in which the carbon black particles are uniformly distributed to form a stable conductive network. As shown in Figure 9b, during the stretching stage, the gap between the carbon black particles increases with the increase in strain, resulting in a change of the conductive network structure. In the unloading stage, when the external force is removed, the spacing between the fillers shrinks and the conductive network structure gradually recovers, as shown in Figure 9c. The Krauss model was used to evaluate the composite tensile-unloading resistance response:

(2) $$N_{1}=\frac{N_{0}}{1}+\left(\frac{\varepsilon }{\varepsilon_{c}}\right){\left(\frac{\varepsilon }{\varepsilon_{c}}\right)}^{2m},$$

(3) $$\rho \propto {\left(N\right)}^{{-n}_{\varepsilon }},$$

(4) $$\frac{∆R}{R_{0}}=\frac{\rho }{\rho_{0}}{\left(\varepsilon +1\right)}^{2}-1={\left(\varepsilon +1\right)}^{2}{\left[{\left(1+{\left(\frac{\varepsilon }{\varepsilon_{c}}\right)}^{2m}\right)}^{-1}\right]}^{{-n}_{\varepsilon }}-1,$$

where N_1_ is the total number of interparticle connections per volume during stretching, N_0_ is the initial number of interparticle connections per volume, m is a constant related to the network structure, ε_c_ is a constant for yield strain, and n_ε_ is the scaling index. Equation (4) better characterizes the resistance–strain relationship in the tensile stage of the composite. During the release process, fracture and destruction of the conductive network occur simultaneously. Some of the disrupted conducting networks are irreversible, resulting in a higher resistance than the initial value. The following equation is employed to describe the change in the number of interparticle N_2_ connections during the release phase due to the complex reconfiguration of the interparticle connections and the irreversible conductive network.

(5) $$N_{2}\left(t\right)=N_{0}\left(k_{1}-k_{2}e^{-\mathrm{Kt}}\right)=N_{0}\left(k_{1}-k_{2}e^{-K\frac{\varepsilon }{έ}}\right),$$

where k_1_, k_2_ and K are constants associated with the process of reformation of interparticle connections, and έ is the strain rate. From Equations (3)–(5), the $\frac{∆R}{R_{0}}$ of the release phase is

(6) $$\frac{∆R}{R_{0}}={\;\left(\varepsilon +1\right)}^{2}{\left[{\left(1+{\left(\frac{\varepsilon }{\varepsilon_{c}}\right)}^{2m}\right)}^{-1}+k_{1}-k_{2}e^{-K\frac{\varepsilon }{έ}}\right]}^{{-n}_{\varepsilon }}-1.$$

The experimental results and theoretical predictions of the resistance strain of CB-KH550/VMQ composites during the loading–unloading cycle are shown in Figure 9d,e, which indicate that the model can describe the experimental data well. The fitting parameters are shown in Table 1.

For CB/VMQ and CB-KH550/VMQ composites, the different values of m indicate that the conductive network structures of the two composites are different, and n_ε_/ε_c_ is consistent with the variation rule of GF values. The reconfiguration process between the particles of the conductive network structure is affected by k_1_, k_2_, and K, which leads to changes in the resistance-strain response.

## 3. Materials and Methods

### 3.1. Materials

Methyl vinyl silicone rubber (VMQ): grade 110-2s, density 1.1 g/cm^3^, molecular weight 6.2 × 10^5^ g/mol, Nanjing Dongjue Silicone Co., Ltd., Nanjing, China. Carbon black (CB): oil absorption value 330 m^2^/g, particle size 12 nm, Chengdu Organic Chemical Co., Ltd., Chengdu, China. Silicon dioxide (SiO_2_): purity > 99.8%, specific surface area 300 m²/g, particle size 7–40 nm, Shanghai McLean Biochemical Technology Co., Ltd., Shanghai, China. Hydroxyl silicone oil (HPMS): analytically pure, Anhui Aida Silicone Oil Co., Ltd., Bengbu, China. Dicumyl peroxide (DCP): analytically pure, Jiangsu Qiangsheng Chemical Co., Ltd., Changshu, China. Ethyl acetate (EA): analytically pure, Tianjin Beichen Fangzheng Reagent Factory, Tianjin, China.

### 3.2. Preparation of CB-KH550/VMQ Composites

**Figure 10:**
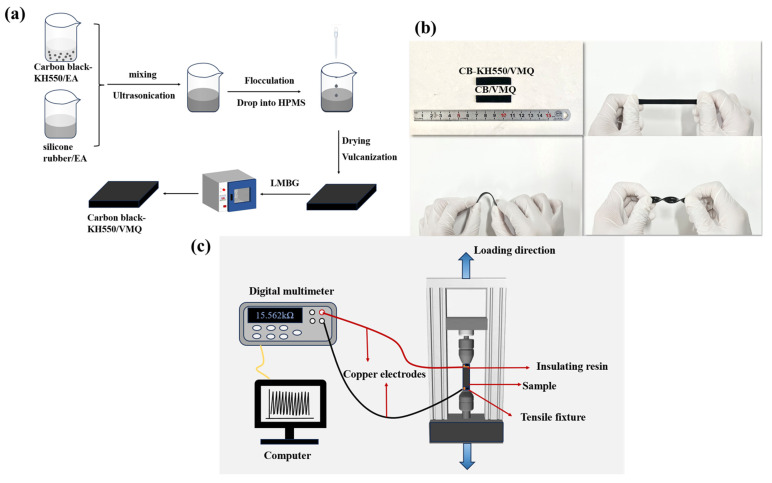
(**a**) Schematic diagram of the preparation of conductive silicone rubber composites; (**b**) images of the specimens under pristine, tensile, flexural, and torsional members; (**c**) schematic diagram of the resistance-strain response testing device.

The CB/VMQ and CB-KH550/VMQ composites were prepared using a solution method. The preparation process is shown in Figure 10a; the experimental ingredients were formulated as shown in Table 2. The process was as follows: (1) CB, SiO_2_ and KH550 were mixed in 200 mL ethyl acetate (EA) and sonicated at 100 W for 1 h to obtain the modified CB dispersion; (2) 4 g of unvulcanized silicone rubber was added into 200 mL EA and mechanically stirred for 1 h to obtain the silicone rubber dispersion; (3) the HPMS, CB, and VMQ dispersions were mixed and sonicated at 100 W for 30 min to obtain the composite; and (4) the temperature was increased to 50 °C, DCP was added to the composite, the solvent was removed with a vacuum, and the resulting mixture was vulcanized at 10 Mpa and 170 °C for 10 min to obtain a CB-KH550/VMQ composite. Finally, the vulcanization molding was followed by 4 h of two-stage vulcanization at 200 °C under atmospheric pressure. Figure 10b shows the photographs of the composites in the original, tensile, flexural, and torsional conditions.

### 3.3. Characterization

The dispersion of CB in the VMQ matrix was observed using scanning electron microscopy (SEM, TESCAN, Brno, Czech Republic). The interfacial interaction between VMQ and CB was analyzed using Fourier Transform Infrared Spectroscopy (FTIR, Bruker Tensor 27, Karlsruhe, Germany) in the wave number range 600~3200 cm^−1^. The interfacial effects of the composites were analyzed using a Raman spectrometer (Raman, Renishaw invia, Ilford, UK) with a laser wavelength of 532 nm and a wave number range of 1000–1800 cm^−1^. The surface elements of composite materials were studied using X-ray photoelectron spectroscopy (XPS, Thermo Scientific K-Alpha, Waltham, MA, USA). The resistance of the composites was measured using a 34410A digital multimeter (Keysight Technologies, Inc., Santa Rosa, CA, USA), and the composites were intercepted into strips of 40 mm × 10 mm × 1 mm, and three sets of 60 s resistance values were taken as the average value. The formula for the conductivity of the composite material is shown below:

(7) σ=1/ρ=L/RS,

where σ is the conductivity (S/m), ρ is the resistivity (Ω-m), L is the length of the composite (m), R is the volume resistance (Ω), and S is the cross-sectional area of the composite (m^2^). An electronic universal testing machine (DDL10, Changchun Testing Machine Research Institute Co., Ltd., Changchun, China) was used to conduct tensile tests to test the mechanical properties of dumbbell-shaped composites, with a tensile rate of 200 mm/min^−1^, and three sets of experimental data were tested to take the average value. The composites (40 mm × 10 mm × 1 mm) were fixed on an electronic universal testing machine to perform resistance-strain response tests under dynamic cyclic loading, and the resistance signals varied during the tests were collected using a digital multimeter. The schematic diagram of the experimental test setup is shown in Figure 10c.

## 4. Conclusions

In this paper, the shoulder peak phenomenon in the resistance response signal of the composite material is eliminated by adding KH550, and the following conclusions are drawn:

(1) The percolation thresholds of CB/VMQ and CB-KH550/VMQ composites are 3.51 wt% and 3.39 wt%, respectively, and both composites form three-dimensional tunneling conductive networks;
(2) The addition of KH550 increased the tensile strength, elongation at the break and Young’s modulus of the composites. The tensile strength, elongation at the break and Young’s modulus were increased from 6.12 MPa, 803.7% and 0.75 MPa to 8.58 MPa, 961.7% and 0.81 MPa, respectively;
(3) Compared with the CB/VMQ composites, the shoulder peak phenomenon was not observed in the resistance-strain response properties of CB-KH550/VMQ composites. Meanwhile, the reason for the disappearance of the shoulder phenomenon was explained by the hysteresis effect and the comparison of SEM images and EDS energy spectra before and after stretching;
(4) An analytical model of resistance-strain response was developed, and the comparison of experimental results and theoretical predictions shows that the model can better characterize the relationship between resistance and strain in the composite material during the tensile-unloading stage.

## Figures and Tables

**Table 1:** Parameters of the fitted strain curves of the two composites.

Filler	m	$\varepsilon_{c}$	$n_{\varepsilon }$	k_1_	k_2_	K
CB-KH550	1.834	0.047	0.283	0.119	0.177	0.134
CB	48.127	67.158	25.225	0.998	0.998	0.043

**Table 2:** The formula of two composites (wt%).

Materials	Content
VMQ	100
CB	Variable X (X = 0, 1, 2, 3, 4, 5, 6, 7, 8)
KH550	Variable Y (Y = X/4)
SiO_2_	20
HPMS	4
DCP	2

## Data Availability

The data presented in this study are available from the corresponding author upon reasonable request.
